# The Influence of Maternal-Foetal Parameters on Concentrations of Zonulin and Calprotectin in the Blood and Stool of Healthy Newborns during the First Seven Days of Life. An Observational Prospective Cohort Study

**DOI:** 10.3390/jcm8040473

**Published:** 2019-04-07

**Authors:** Beata Łoniewska, Dagmara Węgrzyn, Karolina Adamek, Mariusz Kaczmarczyk, Karolina Skonieczna-Żydecka, Grażyna Adler, Agata Jankowska, Izabela Uzar, Agnieszka Kordek, Marta Celewicz, Igor Łoniewski

**Affiliations:** 1 Department of Neonatal Diseases, Pomeranian Medical University, 70-111 Szczecin, Poland; dagpak@poczta.onet.pl (D.W.); karolaadamek1@gmail.com (K.A.); agatajankowska@hotmail.com (A.J.); agkordek@pum.edu.pl (A.K.); 2 Department of Clinical and Molecular Biochemistry, Pomeranian Medical University, 70-111 Szczecin, Poland; mariush@pum.edu.pl; 3 Department of Biochemistry and Human Nutrition, Pomeranian Medical University, 71-460 Szczecin, Poland; karzyd@pum.edu.pl (K.S.-Ż.); sanprobi@sanprobi.pl (I.Ł.); 4 Department of Studies in Antropogenetics and Biogerontology, Pomeranian Medical University, 71-210 Szczecin, Poland; grazyna.adler@pum.edu.pl; 5 Department of General Pharmacology and Pharmacoeconomics, Pomeranian Medical University, 71-230 Szczecin, Poland; uzari@wp.pl; 6 Department of Obstetrics and Gynecology, Pomeranian Medical University, 70-111 Szczecin, Poland; mk.celewicz@gmail.com

**Keywords:** newborn, zonulin, calprotectin, gut barrier, caesarean section, antibiotic, body weight, Body Mass Index (BMI)

## Abstract

Background: It can be hypothetically assumed that maternal and perinatal factors influence the intestinal barrier. Methods: The study was conducted with 100 healthy, full-term newborns breastfed in the first week of life, with similar analyses for their mothers. Zonulin and calprotectin levels were used as intestinal permeability markers. Results: The median (range) zonulin concentrations (ng/mL) were in mothers: serum, 21.39 (6.39–57.54); stool, 82.23 (42.52–225.74); and newborns: serum cord blood, 11.14 (5.82–52.34); meconium, 54.15 (1.36–700.65); and stool at age seven days, 114.41 (29.38–593.72). Calprotectin median (range) concentrations (µg/mL) in mothers were: stool, 74.79 (3.89–211.77); and newborns: meconium, 154.76 (6.93–8884.11); and stool at age seven days 139.12 (11.89–627.35). The use of antibiotics during pregnancy resulted in higher zonulin concentrations in umbilical-cord serum and calprotectin concentrations in newborn stool at seven days, while antibiotic therapy during labour resulted in higher zonulin concentrations in the stool of newborns at seven days. Zonulin concentrations in the stool of newborns (at seven days) who were born via caesarean section were higher compared to with vaginal birth. With further analyses, caesarean section was found to have a greater effect on zonulin concentrations than prophylactic administration of antibiotics in the perinatal period. Pregnancy mass gain >18 kg was associated with higher calprotectin concentrations in maternal stool. Body Mass Index (BMI) increase >5.7 during pregnancy was associated with decreased zonulin concentrations in maternal stool and increased calprotectin concentrations in stool of mothers and newborns at seven days. There was also a negative correlation between higher BMI increase in pregnancy and maternal zonulin stool concentrations and a positive correlation between BMI increase in pregnancy and maternal calprotectin stool concentrations. Conclusion: Maternal-foetal factors such as caesarean section, antibiotic therapy during pregnancy, as well as change in mother’s BMI during pregnancy may increase intestinal permeability in newborns. Changes in body mass during pregnancy can also affect intestinal permeability in mothers. However, health consequences associated with increased intestinal permeability during the first days of life are unknown. Additionally, before the zonulin and calprotectin tests can be adopted as universal diagnostic applications to assess increased intestinal permeability, validation of these tests is necessary.

## 1. Introduction

The intestinal barrier provides highly selective protection of the human body against the environment. It has several facets which include: intestinal bacteria (so-called “intestinal microbiota”); a protective mucus layer containing e.g., mucins; intestinal epithelium (epithelial cells with tight junctions); and cells of the immune system and enteric nervous system (ENS) [1]. The intestinal barrier also includes vascular endothelial cells along with enteric glial cells and pericytes that form the intestinal-vascular barrier [2]. The intestinal barrier performs important physiological functions, for example: (a) absorption: transmembrane transport of liquids, electrolytes and micro- and macroelements; (b) protection: prevention of translocation i.e., entry of various toxic substances and pathogens from the gastrointestinal lumen into the bloodstream as well as to the liver and spleen; (c) transmission of signals to other cells and organs (e.g., via the brain-gut axis). 

A very important function of the intestinal barrier is participation by/in the regulation of the immune and endocrine systems [1]. Disruption of these may lead to increased permeability of the intestinal barrier and be a cause of, or a factor in, aggravation of various chronic diseases, such as irritable bowel syndrome (IBS) [3,4], functional dyspepsia (DC) [5], metabolic syndrome [6], inflammatory bowel disease (IBD) [7], and autoimmune diseases, e.g., rheumatoid arthritis (RA) [8] or autoimmune hepatitis [9].

Diagnostic methods assessing intestinal barrier function based on molecular evaluation of proteins involved with intestinal barrier structure are of great interest; in particular the determination of zonulin and calprotectin in blood and faeces.

Zonulin is an analogue of a cholera toxin (zonula occludens toxin-ZOT), is an established marker of intestinal permeability, and was described more than 20 years ago [10,11,12,13]. It is claimed that it has potential value in the assessment of the paracellular “gateway” in the intestines [14,15,16,17,18,19]. This 47–65 kDa single protein is synthesized in the liver and epithelial cells and can be isolated from a membrane complex (claudin-occludin-guanylate kinase-like zonula occludens (ZO) proteins 1, 2, and 3) which forms tight junctions (TJ) in the apical part of intestinal endothelium [20,21]. Zonulin is also known as pre-HP2, due to its identity to the precursor of haptoglobin-2 [22].

Zonulin is one of the main factors which secures adequate action of the “gut gateway” mechanism by reversibly influencing the tightness of TJs [23,24,25,26], influencing incorporation of proteinase-activating receptor 2 (PAR2) and epidermal growth factor receptor (EGFR), and allowing molecules exceeding approx. 3.5 kDa to cross the intestinal barrier [22,27]. Experimental studies have suggested that a rise in zonulin concentrations parallels increased intestinal permeability [20] and has been demonstrated in *i.a.* an animal model of necrotizing enterocolitis [28,29]. Further, clinical investigations have shown that patients suffering from factors known to give low-grade inflammation (such as type 2 diabetes, celiac disease and obesity) present increased zonulin concentrations [1,30,31,32,33,34,35,36]. The same is true for diseases with known autoimmunological pathomechanisms (e.g., Crohn’s disease, type 1 diabetes) [20,21,37,38]. 

The human homologue of calprotectin is a 24 kDa dimer [39] and is formed by the protein monomers S100A8 (10,835 Da) and S100A9 (13,242 Da). This complex constitutes up to 60% of soluble cytosolic proteins in human neutrophils [12,40]. In addition, it occurs in monocytes, macrophages and epithelial cells. Therefore, faecal calprotectin (FC) content may be proportional to the number of neutrophils migrating through the gastric and intestinal mucosa and may be associated with inflammatory diseases of the gastrointestinal tract. Elevated FC concentrations have been described in IBDs such as Crohn’s disease and ulcerative colitis, and can be used to assess the extent of inflammation in these patients [14,41]. Recently, FC has been found to be associated with allergy to cow-milk proteins and atopic disease, as well as to gastrointestinal disorders [18,19,42,43]. The diagnostic value of FC during infancy is also of increasing interest [20,44]. A higher content of FC in infancy may result from increased intestinal permeability, the formation of intestinal microbiota and/or response to food antigens, as well as perhaps from intestinal colonization by commensal microbes. It is thought that there is a significant correlation between calprotectin concentrations in gut lavage fluid and intestinal permeability, and some have suggested that increased intestinal permeability may result from increased trans-epithelial migration of neutrophils [28,45,46,47]. However FC levels are also high in healthy newborns [38].

Environmental factors related to disturbances in the structure and function of the intestinal barrier include, for example, inadequate diet, a diet rich in animal fats and simple sugars, physical and mental stress, hyperglycemia, drugs (antibiotics, non-steroidal anti-inflammatory drugs, proton-pump inhibitors), gastrointestinal infections and/or upper respiratory tract infections [48]. The aim of our study (with full-born, healthy newborns and their mothers) was to determine zonulin levels in maternal blood and stool before delivery and in umbilical cord blood and newborn meconium as well as stool at age seven days; and calprotectin levels in maternal stool before delivery, newborn meconium and stool at age seven days. We also checked to see if there were any associations between levels of these two markers of intestinal permeability (zonulin and calprotectin) and such factors as: maternal and child body mass, type of delivery and antibiotic treatment. 

## 2. Material and Methods

This observational prospective cohort study was conducted with 100 healthy, full-term newborns born consecutively at the Department of Obstetrics, Gynecology and Neonatology at the Pomeranian Medical University/Independent Public Clinical Hospital No. 2 in Szczecin during the period from March 2015 to April 2016, together with analyses of their mothers (100 women). The total number of deliveries at the Clinic during this period was 1917.

**Figure 1:**
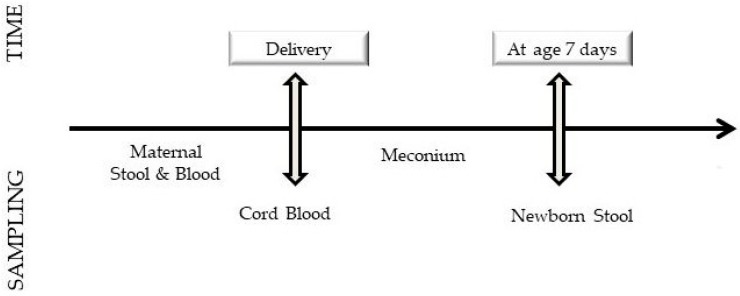
Study plan.

The plan of the study is shown in Figure 1. 

After the initial recruitment of patients carried out by obstetricians at the clinic (women reported due to the beginning of labour or with planned surgical termination of pregnancy), the selection of patients for the study was conducted by an obstetrician (M.C.) or a neonatologist (K.A.). 

After reporting to the emergency department of the hospital’s maternity ward an interview was conducted, a physical examination was performed, height and weight were measured, and foetal and cardiotocography ultrasound were performed. Criteria for inclusion in the study were:

(a) good mother’s health.
(b) gestational age (37–42 weeks).
(c) single pregnancy.
(d) pregnancy control by an obstetrician.
(e) exclusion of a chronic disease (autoimmune diseases including type 1 diabetes, pregnancies complicated by gestational diabetes (GDM) 2, The hemolysis, elevated liver enzymes, low platelet count (HELLP) syndrome).
(f) no pregnancy complications except for infections not requiring hospitalization.
(g) no current infection.
(h) no use of alcohol or psychoactive substances during pregnancy.
(i) absence of foetal defects or symptoms suggestive of infection of the foetus or hypoxia.

The correct course of pregnancy was based on the interview and the pregnancy history. Gestational age was calculated first using the Negelee rule and then corrected based on foetal ultrasound results. After meeting the inclusion criteria, the obstetrician (M.C.) or neonatologist (K.A.) provided detailed information about the patients and the participants of the study signed informed consent to participate in the clinical trial, with the woman giving the right to consent only for certain elements of the study (described below).

After admission to the delivery room, a cannula was placed into a vein through which blood was collected for the analysis of zonulin concentrations. During the first stage of delivery, women who agreed also gave stool for examination. The post-natal state of the newborn was assessed according to the Apgar scale and blood was collected from the umbilical vein to determine concentrations of C-reactive protein, Interleukin-6 and zonulin. The first stool (meconium) of the newborn, and then the stool given at age seven days was collected and secured to determine concentrations of zonulin and calprotectin.

Exclusion criteria were:

(a) premature rupture of membranes (PROM) > 18 h.
(b) maternal infection, congenital infection (clinical signs of infection and/or elevated C-reactive protein (>5 mg/L), Interleukin 6 (>30 pg/mL).
(c) congenital malformations.
(d) asphyxia (Apgar < 8 after 3 min of life, umbilical vein blood pH < 7.2).

No women and no newborns previously qualified for the study met the exclusion criteria. A description of the study group is shown in Table 1.

Written informed consent was obtained from all legal guardians. Study protocol was approved by the Pomeranian Medical University Bioethics Committee (resolution No. KB-0012/55/14 of 30/06/2014) in compliance with the Helsinki Declaration (seventh revision).

The birth weight of the newborns was found to be in the range of 2140 to 4960 g (mean 3427 ± 455 g). On the basis of Fenton centile charts, newborns were initially divided into eutrophic (10–90 centile), hypo- (<10 centile) and hypertrophic (>90 centile) groups. However, due to low numbers in the extreme groups, for statistical calculations the following groups were used: a birth weight between the 15th and 85th centile in relation to foetal age (71% of the cohort studied); with body mass below the 15th centile (15% of the cohort studied); and body mass over the 85th centile (14% of the cohort studied). All newborns in the first week of life were breastfed.

### 2.1. Immunoenzymatic Studies

Immediately after delivery, 5 mL of blood was collected from the umbilical vein into a tube containing Ethylenediaminetetraacetic Acid (EDTA), and was centrifuged for 3 min at 3000–4000 revs/min. A total of 1 mL of serum was transferred to an Eppendorf tube and stored for approximately 24 h at −20 °C until zonulin concentration was determined. The mother’s blood, taken before delivery, was handled identically.

For the determination of zonulin and calprotectin concentrations, the mother’s stools were used from the first stage of delivery, the meconium which the child gave spontaneously within 24 h of life and the stool given on the seventh day of the newborn’s life. The newborn stool was taken from the diaper (using a standardized Stool Sample Application System (SAS) kit from Immundiagnostik, Bensheim, Germany) by trained personnel according to the manufacturer’s procedure. 

Stool was stored for not longer than 3 months at −20 °C until analysis. For analysis, 15 mg of stool was defrosted to room temperature, and analysed following the manufacturer’s protocol (Immundiagnostik, Bensheim, Germany). Absorbance was measured at 450 nm against a 620 nm reference wavelength. Zonulin and calprotectin concentrations were estimated based on a four-parameter logistic regression algorithm. Concentrations of faecal zonulin and calprotectin and serum zonulin were determined by immunoenzymatic methods using commercial Enzyme-Linked Immunosorbent Assay (ELISA) tests (Immunodiagnostik). Zonulin assays were carried out at the Laboratory of Antropogenetics and Biogerontology, and calprotectin assays at the Department of Biochemistry and Human Nutrition, both at the Pomeranian Medical University. If the absorbance was outside of standard curve, the sample was not included in subsequent analyses. As the dilution factor for zonulin was 50, and for calprotectin 2500, and the highest standard concentrations were 16 and 840 ng/mL respectively—we assumed that the highest concentrations we were able to measure were 800 ng/mL and 2100 µg/mL respectively.

### 2.2. Statistical Analyses

Whether variable distributions conformed with normal distributions was examined using Komogorov–Smirnov tests. Due to significant deviations from normality, non-parametric tests were used for most variables: for intergroup comparisons for unpaired variables: Mann–Whitney or Kruskal–Wallis tests were used; for paired variables: Wilcoxon signed rank tests. Spearman coefficients were used to analyse correlations. The following effect sizes were calculated: For Mann–Whitney and Wilcoxon signed rank tests the r value ($\frac{Z}{\sqrt{n}}$) was used, where *z* is the z-score, *n*, total number of observations; for Kruskal–Wallis tests epsilon-squared estimates of effect size were calculated ($\frac{H}{(n^{2}-1)/\left(n+1\right)}$), where *H* is the Kruskal–Wallis H statistic, *n* is total number of observations. To assess relationships between independent and dependent variables multiple linear regression was used with residual analysis for the testing of assumptions of linear regression. All calculations were performed with commercial software (Statistica; Dell Inc., Palo Alto, California, USA, version 13, software.dell.com and Microsoft Excel), and an alpha value of 5% was assumed as the level of significance.

## 3. Results

### 3.1. Zonulin and Calprotectin Concentration

**Figure 2:**
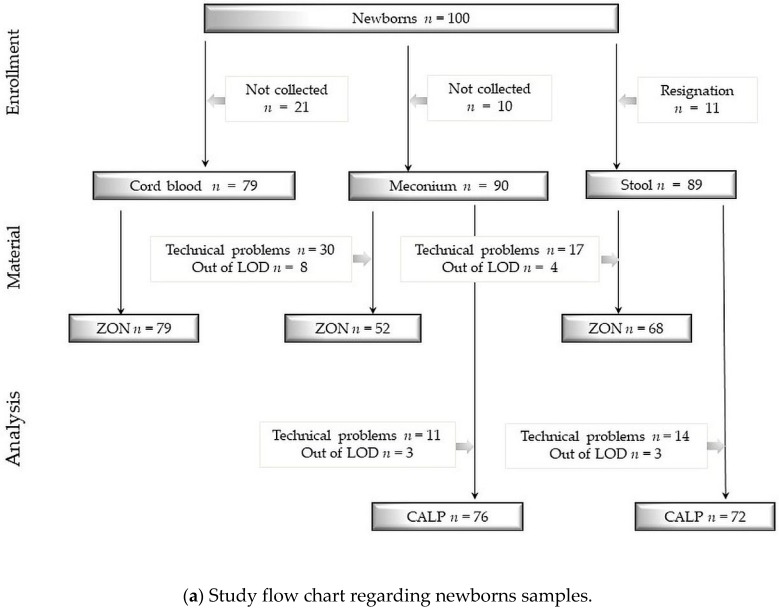

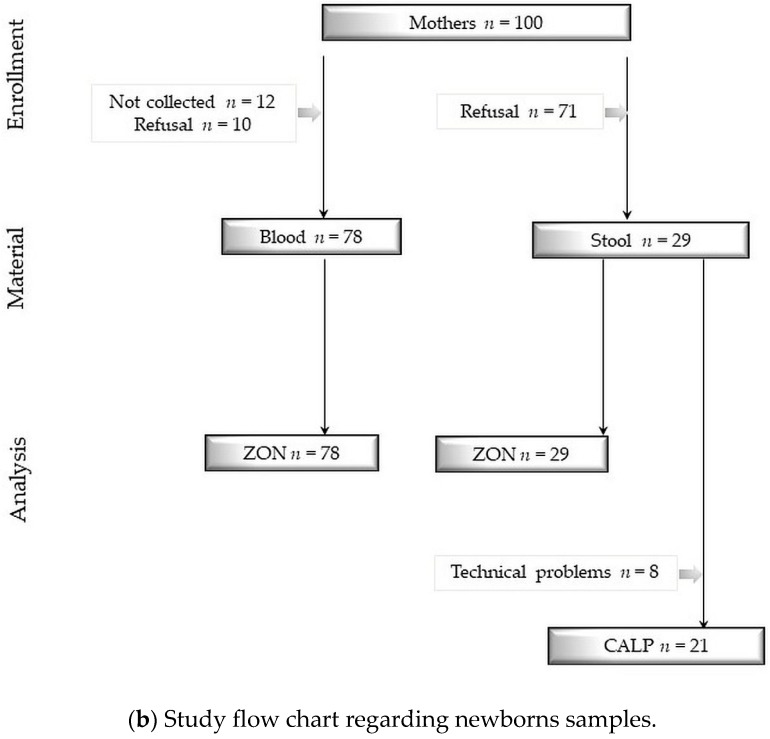
(**a**) Study flow chart regarding newborns samples. (**b**) Study flow chart regarding newborns samples. Study flow charts. Legend: ZON: zonulin, CALP: calprotectin, LOD: limit of detection, numbers and reasons for abandoned samples are listed in frames. Technical problems were sometimes caused due to a small volume of collected stool specimen. Determination limit for zonulin concentration = 800 ng/mL and for calprotectin concentration = 2100 μg/mL.

We obtained 79 samples of cord blood, 90 samples of meconium, 89 samples of neonatal stool, 78 samples of maternal blood and 29 samples of maternal stool. Detailed information concerning samples and analysis are shown in Figure 2a,b. 

Zonulin concentrations are shown in Table 2. In cord blood these were 48% lower than in maternal blood, and these were positively correlated. The highest zonulin concentrations were found in stool at seven days, which were 111% higher than in meconium and 39% higher than in maternal stool. zonulin concentrations in meconium were 34% lower than in maternal stool. Zonulin concentrations in stool and meconium of newborns were positively correlated with each other. Zonulin concentrations in newborns and meconium stool were higher than in umbilical-cord blood by 927% and 386%, respectively. Maternal blood zonulin concentrations were 74% lower than maternal stool concentrations and 60% and 81% lower, respectively, than in meconium and in newborn stool. Zonulin concentrations in maternal blood negatively correlated with that in newborn stool.

Calprotectin concentrations are presented in Table 3. Calprotectin concentrations in maternal stool were lower by 51% than in meconium and by 46% than in newborn stool at seven days of age. A positive correlation was found between calprotectin concentrations in meconium and in newborn stool. There was no correlation between zonulin and calprotectin concentrations.

### 3.2. The Influence of Antibiotic Therapy in the Mother on Zonulin and Calprotectin Concentrations.

The use of antibiotics during pregnancy resulted in higher zonulin concentrations, by 26%, in umbilical-cord serum, while antibiotic therapy during delivery resulted in higher zonulin concentrations, by 45%, in newborn stool at seven days of age. The use of antibiotic therapy during pregnancy was associated with higher, by 81%, calprotectin concentrations in newborns at seven days of age (Table 4 and Table 5).

### 3.3. Zonulin and Calprotectin Concentrations Depending on Type of Labour

Zonulin concentrations in umbilical cord blood and newborn stool at seven days of age, in newborns who were born via caesarean section, were 20% and 54% respectively higher compared to from those with natural births. No influence was found of delivery type on calprotectin concentrations in newborn stool. Results are shown in Table 6. To assess which factor (delivery through caesarean section or administration of antibiotics) affected the concentration of zonulin, the antibiotic group was divided into two subgroups—newborns born with or without caesarean section (who received cefazolin and ampicillin for the prophylaxis of *Streptococcus agalactiae* infection). It was observed that in newborns born through caesarean section zonulin concentrations in stool in the seventh day of life were higher by 66% (post hoc *p* = 0.026) compared to the group that did not receive antibiotics and by 27% (post hoc *p* = 0.049) relative to the group that received prophylactically ampicillin.

However, there were no significant differences in stool zonulin among newborns delivered naturally, with or without ampicillin (post-hoc *p* = 1.0; see Table 7). Multiple regression was carried out to investigate whether the delivery method and/or use of antibiotics could significantly predict zonulin concentrations in stool (at age seven days). Results indicated that the optimal model was a significant predictor of zonulin, F(2,65) = 5.04, *p* = 0.009. Whereas method of delivery contributed significantly to the model (β coefficient = 80.2, *p* = 0.017), the use of antibiotic during delivery was not a significant predictor of zonulin (β coefficient = 17.0, *p* = 0.690).

### 3.4. Zonulin and Calprotectin Concentrations and Maternal Body Mass

Zonulin (ng/mL) and calprotectin (µg/mL) concentrations according to maternal body mass are presented in Supplementary Tables S1–S8. In mothers with body mass increase >18 kg, a higher, by 448%, calprotectin concentration in stool was observed compared to that in mothers whose mass gain during pregnancy was <12 kg (median 98.75 µg/mL, range 74.79–211.77 µg/mL vs. median 18.0 µg/mL, range 17.6–24.31 µg/mL, *p* = 0.047, post hoc test, respectively). In mothers whose BMI increased by more than 5.7 during pregnancy, zonulin stool concentrations were 36% lower (median 72.96 ng/mL, range 42.52–225.74 ng/mL vs. median 114.56 ng/mL, range 75.93–118.70 ng/mL; *p* = 0.004; effect size = −0.55) and calprotectin concentrations were 449% higher (median 98.75 µg/mL, range 38.30–211.77 µg/mL vs. median 18.00 µg/mL, range 3.89–121.13 µg/mL; *p* = 0.003; effect size = 0.66) compared to women who had a BMI increase in pregnancy of <5.7. Calprotectin concentrations in newborn stool at seven days, in which the mothers had a BMI increase in pregnancy of >5.7, were 34.9% higher (median 153.35 µg/mL, range 26.58–571.09 µg/mL vs. median 113.67 µg/mL, range 23.27–627.35 µg/mL; *p* = 0.03; effect size = 0.26) in comparison with newborns of mothers with a BMI increase <5.7. There was also a negative correlation between higher BMI increase in pregnancy and maternal zonulin stool concentrations (R = −0.63, *p* = 0.0004) and a positive correlation between BMI increase in pregnancy and maternal calprotectin stool concentrations (R = 0.59; *p* = 0.006).

### 3.5. The Effect of Birth Weight on Zonulin Concentrations

No effects of birth weight of newborns on zonulin and calprotectin concentrations in the stools of mothers and newborns were found (Supplementary Tables S9 and S10).

## 4. Discussion

To our knowledge, this is the first study in which the influence of maternal-foetal factors on zonulin and calprotectin concentrations in blood serum and in mother’s and newborn’s stool and meconium has been investigated. To carry out further investigations on this topic, concentrations of the tested markers in mothers and newborns should be analysed every day during the first week of life.

Use of these markers in the perinatal period could be of great practical importance because these provide non-invasive methods of assessing the functional state of the intestinal barrier: zonulin is considered a marker of increased permeability, and calprotectin as a marker for intestinal inflammation. The development of the intestinal barrier from birth, and maybe even in intrauterine life, could potentially affect not only the development of the child, but also the development of various diseases in adulthood, for example metabolic disorders, inflammatory bowel diseases and food hypersensitivity [49].

Zonulin is one of the proteins that regulates the function of tight junctions (TJ) between intestinal epithelial cells [21], which determines paracellular transport in the gut. The physiological and pathophysiological significance of transport between foetal intestinal epithelial cells, also referred to as “gut permeability,” is still not well understood. It is known that this phenomenon is important not only in preventing translocation of unwanted molecules from the gastrointestinal tract (e.g., pathogenic bacteria, toxins), but also allows the transport of compounds and molecules necessary for child development and modulation of the intestinal immune system ((Gut Associated Lymphoid Tissue (GALT)) [50]. The development of this system begins in the 10th week of foetal life and basically ends at a variable endpoint [51].

Amniotic fluid that has contact with the intestinal epithelium plays an important role in its formation [52]. One of the main factors which causes increased production of zonulin is intestinal or amniotic microbiota [49]. Zonulin is secreted into the gastrointestinal lumen where it stimulates protease-activated receptors (PAR) and epidermal growth factor receptors (EGFr), which induce a complex process of “opening” TJs causing increased paracellular permeability. It can be said that the stool zonulin concentration is a marker for the rate of its production in enterocytes, and the concentration in the blood is related to the transport of this protein from the intestinal lumen into the submucosal layer, between intestinal epithelial cells.

After delivery, there is a reduction in intestinal permeability referred to as “gut closure” [50]. In the case of various animal species the duration of this varies and lasts from a few to several days [53]. In humans, the period necessary for “gut closure” after delivery is unknown (but is prolonged if the breastfeeding delay lasts longer than 30 h) [54], and is thought to be regulated by intrinsic factors, growth factors, hormones and the mother’s food. The “gut closure” process is also associated with an increase in thickness and density of the mucus lining of the intestine, which reduces the uptake of lactoglobulins and macromolecules [55]. Zonulin concentrations in maternal stool, as observed in this study, were higher than in serum, confirming that in the mothers no pathologically-increased intestinal permeability had occurred. A similar relationship was observed in newborns, with a higher concentration of zonulin in meconium than in umbilical-cord blood.

Zonulin concentrations in newborn stool at seven days were higher than in meconium, which indicates increased production of zonulin in the first week of life. In the literature there are no data on zonulin concentrations during this exact period for mothers or newborns. There are, however, two studies which describe zonulin concentrations in newborns. Firstly, Tarko et al. [56] compared zonulin concentrations (by ELISA) in newborn patients (newborns with sepsis, extremely low birth weight, necrotizing enterocolitis, rotavirus infection and abdominal wall defects) with a control group from the second to the 11th day of life. Significantly higher zonulin concentrations were found in rotavirus-infected newborns and with abdominal wall defects (AWD). The range of observed values of zonulin was 2.7 to 4.8 ng/mL in the control group (median 3.5 ng/mL) and 2.0 to 43.2 ng/mL in the group of ill newborns [56]. The range of concentrations in umbilical cord serum in the study presented here was 5.82 to 52.34 ng/mL (median 11.14 ng/mL) and this was higher than in the study by Tarko et al. [56]. Secondly, Saleem et al. [57] observed that zonulin concentrations in newborns born before the 28th week of pregnancy were lower compared to the control group, but zonulin concentrations were not given in the study.

A large variation in zonulin concentrations in umbilical-cord serum may be a consequence of its immunogenic properties. Zonulin is a protein with a molecular mass greater than 5 kDa and can activate the innate immune response and be captured by macrophages and Browicz-Kupffer cells in the liver. For this reason there may be significant differences in serum zonulin concentrations (from undetectable to very high) occurring within minutes or hours [17,31]. The half-life of these molecules in the blood is very variable and ranges from 4 min up to 4 h. Some authors have suggested measuring, instead of zonulin itself, IgA and IgG antibody levels against zonulin as more stable parameters to assess the state of the intestinal barrier [58]. Another reason for the large spread of zonulin concentration measurements might be the use of the ELISA method. However, this method is commonly used in laboratories to assess the integrity of the intestinal barrier.

Scheffer et al. [59] have suggested that the use of the Immundiagnostik ELISA kit, which was designed primarily for the measurement of serum zonulin (pre-HP2) levels, might actually identify a variety of proteins structurally and possibly functionally related to zonulin, suggesting the existence of a family of zonulin proteins as previously hypothesized [23,29], rather than a single permeability-regulating protein. The authors postulated that additional studies were necessary to establish the primary target proteins (zonulin, properdin and/or other structurally similar proteins) detected by this commercially available ELISA kit.

The range of serum zonulin concentrations in mothers was also large (6.39 to 57.54 ng/mL) and consistent with ranges described in other works (e.g., [60]). The literature contains very different values for zonulin concentrations in stool. Malickowa et al. reported (using an ELISA from OrgenTec, Germany) zonulin concentrations in the stool of 61 ± 46 ng/mL [61]. Furthermore, Lamprecht et al. gave 30 ng/mL as a cut-off point [62]. It should be emphasized that zonulin concentration measurements in stool have not previously been reported in women and newborns during the perinatal period. Material collection at this period is very difficult and this may also be the cause of variable results. The meconium is a dense, sticky stool, and its proper collection (with “entrance” necessary to the holes of brushes measuring the right amount of material) poses problems. On the other hand, the stools of newborns at age seven days consist of lumps and fluid that soaks into the diaper, so it is possible to take mostly the lumps, whose very compact consistency also causes difficulties in using the Stool Sample Application System (SAS).

FC concentrations depend on the numbers of neutrophils migrating from the mucous membrane of the stomach and intestines. This may be associated with inflammatory diseases of the gastrointestinal tract, and during infancy may result from increased intestinal permeability, response to food antigens, as well as via colonization of the gut by bacteria. Rhodas et al. have supported this hypothesis finding elevated FC level associated with stool microbiota alterations (reduced *Bifidobacilli*) in babies with colic [63]. Calprotectin concentrations in meconium and newborn stool at day 7 observed in this study were significantly higher than in maternal stool. These observations are consistent with the results of other studies. Healthy full-term and preterm infants, especially younger than 3 months old, have high faecal calprotectin values, similar to those observed in patients with IBD, although with large interindividual variation. No significant differences occur between full-term and preterm newborns at the same postnatal age [64,65]. In our study, no difference was observed between calprotectin concentrations in meconium and in newborn stool at day 7. Baldassarre et al. [66] described in full-term newborns a slight but significant increase in levels at day 7 compared with levels at day 3, the levels then remaining similar for the first month of life. In a study by Lee et al. [67] mean FC concentrations decreased over time from 322 μg/g (0 to 2 months) to 197 μg/g (2 to 4 months) and then to 111 μg/g (at 4 to 6 months). This pattern also occurred within individuals. 

FC reduction with age may indicate a maturation process, including decreased intestinal permeability, in the intestinal mucosa. For adults and newborns over 4 years, a cut-off level of 50 μg/g has been established for diagnostic purposes [14,41]. Therefore, we may be able to define when intestinal mucosa maturation completes by determining when the FC reaches below 50 μg/g. This follows from: firstly, the high calprotectin concentrations observed in the newborn period which may be related to the unusual physiology of the newborn gut. Indeed, a specific pattern of functioning in the first few weeks of life is characterised by, amongst other things, an increased transmucosal leakage, as shown by Walker [68]. As there is no accumulation of calprotectin-rich leucocytes in the healthy mucosa in the first few months of life, the high calprotectin concentrations observed may reflect increase in intestinal permeability leading to transepithelial migration of neutrophils. Intestinal permeability remains significantly higher during the newborn period compared with adulthood. Secondly, gut microbiota establishment may have an effect on calprotectin release, as suggested by Baldassarre et al. [66] and Josefsson et al. [69]. Savino et al. observed higher FC concentration in healthy breast-fed infants with a median age of 51 days (range 13–90) in comparison to formula-fed infants [70]. Results suggest that different ingredients of mother milk (bacteria, hormones, immunestimulating agents) affect development of intestinal barrier and that FC could be a good marker of intestinal barrier permeability.

In our study, a difference of seven days in the maturation of the intestinal barrier did not seem to effect differences in calprotectin concentrations in newborn stool. All newborns included in our study were breastfed for the first week of life. Admittedly, higher FC levels in exclusively breastfed infants, compared with formula-fed infants, aged < 6 months could be consistent with human milk-promoted growth of the intestinal mucosa, even though breast milk has also been shown to promote a reduction in intestinal permeability. Seven days might have been too short a period to find significant differences in calprotectin concentrations in stool. 

In newborns born via caesarean section, umbilical cord blood and stool zonulin concentrations at seven days were significantly higher than in newborns born naturally. This can be assumed to arise from increased permeability of the intestinal barrier after delivery. In Caesarean newborns, zonulin concentrations in meconium, were not significantly greater than in newborns born naturally. That is, increased zonulin stool concentrations at seven days of age was not related to zonulin from the mother nor from foetal life. It can be assumed that this phenomenon may be related to differences in microbiota between newborns born naturally or through caesarean section. No effect was found of delivery method on newborn stool calprotectin concentrations. Campetto et al. and Baldassarre et al. also did not find that delivery method influenced faecal calprotectin in full-term newborns [66,71], whereas Josefsson et al. [69] found a positive correlation with caesarean delivery in preterm infants. Lee et al. [67] reported that FC levels in those born by vaginal delivery were higher than FC levels of caesarean section newborns. 

The gastrointestinal tract becomes rapidly colonized by microorganisms from the delivery environment [72,73]. Natural vaginal delivery provides early contact with the maternal microbiome and is considered crucial for gut maturation, metabolic and immunologic programming and host–microbe homeostasis. Several reports have shown differences in infant faecal microbiomes according to delivery mode and that early colonization patterns can be affected by mode of delivery [73].

Another factor that affects the composition of microbiota is the intake of antibiotics. Antibiotic therapy used during labour caused increased newborn-stool zonulin concentrations at day 7, similarly to that for caesarean section. Umbilical-cord serum concentrations were also higher in case of antibiotics administration during pregnancy. The mothers’ blood zonulin concentrations were not affected by antibiotics, but it can be assumed that increased zonulin concentrations occurred in the foetus under the influence of the mother’s antibiotic therapy. It is assumed that up to 40% of newborns are indirectly exposed to antibiotics during vaginal delivery [72,74]. The consequences of antibiotic therapy in the perinatal period could either result in the emergence of antibiotic resistant strains [75] and/or be indirectly related to the occurrence of asthma and obesity [76]—diseases also associated with early microbiota alterations [77]. Increases in zonulin concentrations in the umbilical-cord blood of mothers who used antibiotics during pregnancy may be associated with deleterious composition of foetal intestinal microbiota. During pregnancy, the foetus has contact with bacteria in foetal membranes, amniotic fluid, placenta and intestines [78]. It seems, however, that caesarean section, which exposes mother and child to many adverse factors (anesthesia, surgery, postoperative pain, use of analgesics, stress, etc.) is a factor which could affect intestinal permeability more than the administration of antibiotics in the perinatal period. It should be noted that the prophylactic use of ampicillin in the perinatal period does not affect intestinal permeability, and in children after caesarean section this is evident both during labour (with increase in zonulin concentration in umbilical cord blood) and on the seventh day after delivery.

The use of antibiotics by a pregnant woman not only affects her microbiome, but also the microbiome of her child which affects, among other things, the immune system at the beginning of life [79]. This may result in secretion of more zonulin in the foetal intestine, resulting in increased concentrations in umbilical-cord blood and, as a consequence, increased foetal intestinal permeability. This hypothesis is supported by the observation that the use of antibiotics during pregnancy was associated with an increased concentration of calprotectin in newborn stool at day 7. Admittedly, faecal calprotectin has previously been found to correlate negatively with antibiotic treatments in this population of very low birth weight infants [69] but in newborns with cystic fibrosis antibiotic treatment resulted in a larger but statistically significant increase in FC [80]. The results of our study, which was carried out in healthy newborns, suggested increased neutrophil activity, probably due to changes in the microbiota in newborns caused by the use of antibiotics during pregnancy.

The obtained results suggest extreme caution when using antibiotics in pregnant women, because they could have long-term consequences for the child. It should also be emphasized that the influence of antibiotic therapy during pregnancy on the foetus and later on for the health of the child is still the subject of scientific debate. In addition, the observed results indicate the need to avoid caesarean delivery, which may take place under the influence of patients’ expectations, without medical indication.

Some Polish researchers have observed positive correlations between serum zonulin concentrations and body mass, body fat percentage, glucose concentrations and the amount of energy supplied, indicating that the concentration of zonulin might be used as a marker for chronic inflammation and increased intestinal permeability [81]. Serum zonulin concentrations are also elevated in women with gestational diabetes (GDM). The cut-off value for these patients was 43.3 ng/mL, with a sensitivity of 88% (95% Confidence Interval (CI): 71–100%) and specificity of 47% (95% CI: 33–58%), suggesting that plasma zonulin concentrations are a possible predictor of this disease [82].

In this study, no associations were found between zonulin or calprotectin concentrations in newborns with maternal mass before pregnancy. However, negative correlations were found between BMI, and maternal body mass gain during pregnancy, with stool zonulin concentrations (but no correlations with serum zonulin). Further, calprotectin concentrations in the mother’s stool correlated positively with an increase in BMI during pregnancy. These results for zonulin are difficult to interpret, partly because zonulin concentrations in maternal stool have not previously been evaluated during the perinatal period. Perhaps hormonal and physiological changes associated with childbirth result in “hypoexpression” of zonulin production in enterocytes and the “sealing” of the intestinal barrier to protect the body against bacteria and toxins from the gastrointestinal tract. Zonulin concentrations in other periods of pregnancy have also not been studied. Maternal zonulin concentrations during the perinatal period were measured mainly to compare with the determinations made in newborns, and were not the main purpose of this work. The observed phenomenon however is very interesting and requires further research, but due to significant methodological limitations is quite difficult to carry out (healthy mothers just before delivery are in general not willing to participate in complicated clinical trials).

The observed increase in calprotectin in newborns at age seven days and in mothers whose BMI increase was greater than 5.7 and body mass increase was greater than 18 kg is consistent with predictions. Calprotectin is considered to be a marker of obesity and metabolic disorders both in adults [83] and in children [84], although pregnancy alone does not affect calprotectin concentrations in maternal stool [85]. However, increased BMI may be associated with the occurrence of inflammation during pregnancy, which may also translate into the occurrence of inflammation in newborns.

Limitations of our study are as follows. (1) There is a risk of recall bias associated with an interview regarding antibiotic intake during pregnancy. Although these data were verified using the records in the pregnancy sheet, the information obtained was subject to this risk of error. (2) Relatively high numbers of abandoned samples were caused by technical problems and circumstances related to the delivery period, including psychological instability of mothers. (3) Maternal diet composition was not analysed. (4) Intestinal permeability was not measured directly, and (5) neither was stool microbiome composition or metabolic function characterised. We therefore could not prove direct relationships between intestinal permeability, the faecal microbiome and FC or zonulin levels and additional research should address these factors. We also did not investigate health consequences associated with increased intestinal permeability during the first few days of life.

## 5. Conclusions

Maternal-foetal factors such as caesarean section and antibiotic therapy during pregnancy, as well as changes in a mother’s BMI during pregnancy may all affect increased intestinal permeability in newborns. Changes in body mass during pregnancy can also affect intestinal permeability in mothers. However, the health consequences associated with increased intestinal permeability during the first few days of life are unknown. Additionally, before the zonulin and calprotectin tests are adopted as universal diagnostic applications to assess increased intestinal permeability, it is necessary to validate these tests. 

## Figures and Tables

**Table 1:** Newborn and mother characteristics.

**Newborns, *n* = 100**				
Gender: Male (%)	56.0%			
Birth weight (g), mean ± standard deviation (SD), (range)	3427 ± 455 (2140–4960)			
≤15th percentile	15%			
≥85th percentile	14%			
**Mothers, *n* = 100**				
Age (years), mean ± SD (range)	27.7 ± 5.3 (18–41)			
Vaginal birth (induction with oxytocin)	39% (21 from 100)			
First pregnancy	60%			
First delivery	62%			
Antibiotic therapy during pregnancy	30%			
Antibiotic therapy during childbirth * (prevention of GBS infection)	81% (20%)			
Median (range) increase in BMI during pregnancy	5.7 (0.58–11.2)			
A. Categories: BMI before pregnancy	<18.5	≤18.5 <25	≥25 <30)	≥30
Percent within each BMI category A.	12%	56%	20%	12% (12%)
B. Categories: BMI before childbirth	<18.5	≤18.5 <25	≥25 <30	≥30
Percent within each BMI category B.	0%	16%	36%	48% (48%)
C. Categories: Weight gain during pregnancy	<12 kg	12–18 kg	>18 kg	
Percent in each weight gain category C.	25%	39%	36%	

BMI: Body Mass Index; *: half an hour before Caesarean section, a woman received intravenous cefazolin at a dose of 2 g. If cervical colonization of *Streptococcus agalactiae* was found or with lack of evidence of no colonization, women received intravenous ampicillin at 2 g which was repeated every 4 h until the child was born.

**Table 2:** Zonulin concentrations (ZON) (ng/mL) in the blood and stool of mothers and newborns, with correlations.

	Umbilical-Cord Blood	Meconium	Newborn Stool	Maternal Stool	Maternal Blood
**Concentration (ng/mL)**					
**ZON** **Median (range)**	*n* = 79	*n* = 52	*n* = 68	*n* = 29	*n* = 78
	11.14 (5.82–52.34)	54.15 (1.36–700.65)	114.41 (29.38–593.72)	82.23 (42.52–225.74)	21.39 (6.39–57.54)
**Differences and correlations**					
**Umbilical-cord blood**	x	*n* = 43	*n* = 57	*n* = 29 vs. *n* = 79	*n* = 79 vs. *n* = 78
		*p* < 0.00001 † (r = 0.70)	*p* < 0.00001 † (r = 0.87)	*p* < 0.00001 ‡ (r = −0.76)	*p* < 0.00001 ‡ (r = −0.55)
**Meconium**	*n* = 43	x	*n* = 36	*n* = 52 vs. *n* = 39	*n* = 52 vs. *n* = 78
	R = −0.07 *p* = 0.640		*p* = 0.0002 † (r = 0.61)	*p* = 0.003 ‡ (r = −0.33)	*p* = 0.000002 ‡ (r = 0.42)
**Newborn stool**	*n* = 57	*n* = 36	x	*n* = 29 vs. *n* = 68	*n* = 68 vs. *n* = 78
	R = −0.08 *p* = 0.564	R = 0.31 *p* = 0.063		*p* = 0.026 ‡ (r = 0.23)	*p* < 0.00001 ‡ (r = 0.86)
**Maternal stool**	*n* = 20	*n* = 14	*n* = 18	x	*n* = 23
	R = 0.27 *p* = 0.257	R = −0.42 *p* = 0.140	R = 0.12 *p* = 0.636		*p* = 0.00002 (r = 0.88) †
**Maternal blood**	*n* = 68	*n* = 45	*n* = 52	*n* = 23	x
	R = 0.27 *p* = 0.025	R = −0.12 *p* = 0.439	R = −0.39 *p* = 0.004	R = 0.18 *p* = 0.398	

R: correlation coefficient, *p*: statistical significance, r: effect size, ‡ Mann–Whitney test, † signed rank Wilcoxon test.

**Table 3:** Calprotectin concentrations (CALP) (μg/mL) in newborn and maternal stool and correlations between these.

	Meconium	Newborn Stool	Maternal Stool
**Concentration (μg/mL)**			
**CALP Median (range)**	*n* = 76	*n* = 72	*n* = 21
	154.76 (6.93–884.11)	139.12 (11.89–627.35)	74.79 (3.89–211.77)
**Differences and Correlations**			
**Meconium**	x	*n* = 64	*n* = 76 vs. *n* = 21
		*p* = 0.179 † (r = 0.17)	*p* = 0.0001 ‡ (r = 0.39)
**Newborn stool**	*n* = 65	x	*n* = 72 vs. *n* = 21
	R = 0.45 *p* = 0.0002		*p* = 0.0005 ‡ (r = 0.36)
**Maternal stool**	*n* = 18	*n* = 16	x
	R = 0.03 *p* = 0.906	R = 0.50 *p* = 0.047	

R: correlation coefficient, p: statistical significance, r: effect size, ‡ Mann–Whitney test. † signed rank Wilcoxon test.

**Table 4:** Effects of antibiotic therapy during pregnancy on zonulin (ZON, ng/mL) and calprotectin (CALP, μg/mL) concentrations.

	Antibiotic Therapy During Pregnancy Median (Range)		
	YES	No	*p*/r
**ZON in the mother’s serum**	*n* = 24	*n* = 54	0.389/0.10
	22.49 (10.26–42.98)	19.78 (6.39–57.54)	
**ZON in umbilical cord blood**	*n* = 24	*n* = 55	0.007/0.30
	13.46 (7.47–52.34)	10.66 (5.82–47.92)	
**ZON in maternal stool**	*n* = 9	*n* = 20	0.795/−0.05
	80.97 (42.52–208.20)	93.17 (42.87–225.74)	
**ZON in meconium**	*n* = 16	*n* = 36	0.117/−0.22
	30.32 (2.13–700.65)	62.42 (1.36–309.2)	
**ZON in newborn stool**	*n* = 21	*n* = 47	0.248/−0.14
	105.23 (55.03–339.46)	125.20 (29.38–593.72)	
**CALP in maternal stool**	*n* = 4	*n* = 17	0.347/0.21
	111.48 (3.89–179.82)	58.49 (13.26–211.77)	
**CALP in meconium**	*n* = 23	*n* = 53	0.218/0.14
	182.29 (19.62–884.11)	137.62 (6.93–866.24)	
**CALP newborn stool**	*n* = 21	*n* = 51	0.002/0.36
	205.87 (56.38–530.60)	113.67 (11.89–627.35)	

*p*—statistical significance, r—effect size.

**Table 5:** Effects of antibiotic therapy during delivery on zonulin (ZON, ng/mL) and calprotectin (CALP, μg/mL) concentrations.

	Antibiotic Therapy During Delivery Median (Range)		
	YES	NO	*p*/r
**ZON in umbilical cord blood**	*n* = 60	*n* = 15	0.071/0.21
	10.87 (5.82–52.34)	14.77 (6.34–30.20)	
**ZON in meconium**	*n* = 38	*n* = 10	0.501/0.10
	54.15 (1.36–700.65)	81.12 (1.93–181.82)	
**ZON in** **newborn stool**	*n* = 52	*n* = 12	0.041/−0.25
	124.77 (29.38–593.72)	85.70 (52.84–173.40)	
**CALP in maternal stool**	*n* = 15	*n* = 6	0.149/-0.31
	83.47 (3.89–211.77)	46.36 (13.26–98.75)	
**CALP in meconium**	*n* = 60	*n* = 12	0.436/−0.09
	163.66 (6.93–884.11)	128.54 (32.39–570.49)	
**CALP in newborn stool**	*n* = 53	*n* = 15	0.162/−0.17
	140.01 (11.89–627.35)	64.15 (35.76–530.60)	

*p*—statistical significance, r—effect size.

**Table 6:** Effects of delivery method on zonulin (ZON, ng/mL) and calprotectin (CALP, μg/mL) concentrations.

	Vaginal Birth Median (Range)	Caesarean Section Median (Range)	*p*/r
**ZON in umbilical cord blood**	*n* = 31	*n* = 48	0.040/0.23
	12.84 (6.34–30.20)	10.68 (5.82–52.34)	
**ZON in meconium**	*n* = 24	*n* = 28	0.283/−0.15
	32.59 (1.93–309.2)	60.63 (1.36–700.65)	
**ZON in the newborn stool**	*n* = 29	*n* = 39	0.002/−0.38
	92.49 (29.38–323.93)	142.46 (50.97–593.72)	
**CALP in meconium**	*n* = 28	*n* = 48	0.481/−0.08
	143.49 (11.43–737.25)	169.73 (6.93–884.11)	
**CALP in newborn stool**	*n* = 30	*n* = 42	0.828/−0.03
	140.09 (35.76–530.60)	139.12 (11.89–627.35)	

*p*—statistical significance, r—effect size.

**Table 7:** Effects of antibiotic therapy on zonulin (ZON, ng/mL) and calprotectin (CALP, μg/mL) concentrations.

	Antibiotic Therapy During Delivery and Delivery Method; Median (Range)			
	N	NAMP	CC	*p*/r
**ZON in the mother’s serum**	*n* = 17	*n* = 13	*n* = 45	0.980/0.0005
	20.58 (12.07–28.66)	21.37 (12.70–44.90)	21.95 (6.39–57.54)	
**ZON in umbilical cord blood**	*n* = 15	*n* = 12	*n* = 48	0.078/0.07
	14.77 (6.34–30.20)	12.92 (7.48–22.15)	10.68 (5.82–52.34)	
**ZON in maternal stool**	*n* = 6	*n* = 6	*n* = 17	0.357/0.07
	110.67 (54.54–225.74)	93.94 (72.96–149.01)	80.11 (42.52–208.20)	
**ZON in meconium**	*n* = 10	*n* = 10	*n* = 28	0.268/0.06
	81.13 (1.93–181.82)	23.01 (3.77–309.20)	60.63 (1.36–700.65)	
**ZON in** **newborn stool**	*n* = 12	*n* = 13	*n* = 39	0.005 †/0.17
	85.70 (52.84–173.40)	88.24 (29.38–323.93)	142.46 (0.97–593.72)	
**CALP in maternal stool**	*n* = 6	*n* = 2	*n* = 13	0.334/0.11
	46.35 (13.26–98.75)	73.10 (26.95–119.25)	83.47 (3.89–211.77)	
**CALP in meconium**	*n* = 12	*n* = 12	*n* = 48	0.734/0.009
	128.54 (32.39–570.49)	157.59 (20.28–737.25)	169.73 (6.93–884.11)	
**CALP newborn stool**	*n* = 15	*n* = 11	*n* = 42	0.333/0.03
	64.15 (35.76–530.60)	165.63 (56.38–235.59)	139.12 (11.8–627.35)	

† N vs. CC 0.027, NAMP vs. CC 0.030, N vs. NAMP 1.0—post hoc analysis; N—vaginal birth without antibiotic, NAMP—vaginal birth and prophylactic treatment with ampicillin, CC—caesarean section and application of cefazolin.

## Supplementary Materials

The following are available online at https://www.mdpi.com/2077-0383/8/4/473/s1, Table S1: Zonulin concentrations in women overweight, with obesity and underweight; Table S2: Calprotectin concentrations in women overweight, with obesity and underweight; Table S3: Zonulin concentrations according to mother’s body weight increase during pregnancy; Table S4: Calprotectin concentrations according to body weight increase of pregnant women; Table S5: Zonulin concentrations according to increase in BMI during pregnancy; Table S6: Concentrations of calprotectin according to increase in BMI in pregnancy; Table S7: Correlations between maternal BMI and Zonulin concentrations; Table S8: Correlations between maternal BMI and calprotectin concentrations; Table S9: Effect of birth weight on Zonulin concentrations; Table S10: Effect of birth weight on calprotectin concentrations.
