# Supplementary material for: The Influence of Maternal-Foetal Parameters on Concentrations of Zonulin and Calprotectin in the Blood and Stool of Healthy Newborns during the First Seven Days of Life. An Observational Prospective Cohort Study
Source: J Clin Med. 2019 Apr 7;8(4):473. doi: 10.3390/jcm8040473 (PMC6517987; doi:10.3390/jcm8040473)
Supplement: Supplementary file 1 [file jcm-08-00473-s001.pdf]

Supplementary Materials: **The influence of maternal-foetal parameters on concentrations of zonulin and calprotectin in the blood and stool of healthy newborns during the first seven days of life. An observational prospective cohort study.**

Supplemental Table 1. Zonulin concentrations (ZON, ng/ml) in women: overweight, with obesity and underweight.

BMI before pregnancy	≤18.5	>18.5	
	Median (range)	Median (range)	p/r
ZON in meconium	56.42 (1.93-148.24)n=8	51.05 (1.36-700.65) n=43	0.595/ 0.07
ZON in newborn stool	132.07 (61.36-337.0) n=8	115.28 (29.38-593.72) n=58	0.992/ -0.001
ZON in umbilical-cord serum	11.83 (6.86-35.74) n=12	11.14 (5.82-52.34) n=65	0.710/ -0.04
ZON in maternal serum	26.89 (12.07-42.25) n=10	20.31 (6.39-57.54) n=66	0.279/ -0.12
ZON in maternal stool	94.95 (57.25-153.17) n=4	81.60 (42.52-225.74) n=24	0.870/ -0.03
BMI before pregnancy	≤25	>25	
ZON in meconium	48.01 (1.36-289.20) n=37	61.77 (10.72-700.65) n=14	0.454 -0.10
ZON in stool	109.36 (52.42-505.51) n=43	128.37 (29.38-593.72) n=23	0.463/ -0.09
ZON in umbilical-cord serum	10.72 (5.82-52.34) n=55	12.87 (5.96-36.57) n=22	0.107/ -0.18
ZON in maternal serum	21.37 (6.39-57.54) n=55	21.95 (10.26-44.90) n=21	0.857/ -0.02
ZON in maternal stool	76.59 (42.52-225.74) n=19	106.09 (48.20-208.20) n=9	0.302/ -0.20
BMI before pregnancy	≤30	>30	
ZON in meconium	69.95 (12.88-122.11) n=47	51.05 (1.36-700.65) n=4	0.713/ -0.05
ZON in newborn stool	118.70 (50.97-505.51) n=58	107.83 (29.38-593.72) n=8	0.992/ 0.001
ZON in umbilical-cord serum	11.07 (5.82-52.34) n=70	14.11 (7.47-22.15) n=7	0.199/ -0.15
ZON in maternal serum	21.40 (6.39-57.54) n=67	18.13 (11.99-44.90) n=9	0.930/ -0.01
ZON in maternal stool	80.11 (42.52-225.74) n=23	100.13 (78.24-188.70) n=5	0.472/ -0.14

p - statistical significance, r-effect size

Supplemental Table 2. Calprotectin concentrations (CALP, µg/ml) in women: overweight. with obesity and underweight.

BMI before pregnancy	≤18.5	>18.5	
	Median (range)	Median (range)	p/r
CALP in meconium	121.60 (6.93-538.91) n=9	154.76 (7.48-884.11) n=64	0.445/0.09
CALP in newborn stool	76.66 (35.76-286.87) n=10	140.00 (23.27-627.35) n=59	0.512/0.08
CALP in maternal stool	14.60 (13.26-179.82) n=3	74.79 (3.89-211.77) n=17	0.459/0.17
BMI before pregnancy	≤25	>25	
CALP in meconium	145.07 (6.93-884.11) n=50	166.99 (7.48-866.24) n=23	0.098/-0.19
CALP in newborn stool	120.82 (26.58-627.35) n=47	151.30 (23.27-571.09) n=22	0.274/-0.13
CALP in maternal stool	74.79 (3.89-211.77) n=13	58.49 (17.60-119.25) n=7	0.812/0.05
BMI before pregnancy	≤30	>30	
CALP in meconium	152.16 (6.93-884.11) n=63	197.82 (82.45-737.25) n=10	0.165/-0.16
CALP in newborn stool	140.17 (23.27-627.35) n=60	120.09 (44.83-203.04) n=9	0.454/0.09
CALP in maternal stool	80.12 (3.89-211.77) n=17	24.31 (18.00-58.49) n=3	0.290/0.24

p - statistical significance, r-effect size

Supplemental Table 3. Zonulin concentrations (ZON, ng/ml) according to mother's mass increase during pregnancy.

Mother's mass increase during pregnancy	<12 kg	12-18 kg	>18 kg	p/epsilon-squared
	Median (range)	Median (range)	Median (range)	
ZON in meconium	46.17 (1.93-124.01) n=11	87.99 (7.96-700.65) n=20	57.25 (1.36-148.24) n=17	0.290/ 0.05
ZON in newborn stool	110.42 (29.38-593.72) n=19	129.67 (55.03-505.51) n=26	105.23 (50.7-369.08) n=19	0.377/ 0.03
ZON in umbilical-cord serum	10.58 (6.86-25.06) n=17	11.17 (7.66-52.34) n=32	11.12 (5.82-47.92) n=25	0.411/ 0.02
ZON in maternal stool	100.13 (75.93-188.70) n=7	111.29 (54.54-225.74) n=13	57.25 (42.52-208.20) n=9	0.030†/ 0.025
ZON in maternal serum	21.37 (10.26-40.74) n=17	21.49 (6.39-42.98) n=30	21.95 (9.28-57.54) n=27	0.935/ 0.002

† <12 vs 12-18: p=1.0; <12 vs >18: p=0.074; 12-18 vs >18: 0.056 (post-hoc test), p - statistical significance, epsilon-squared – effect size

Supplemental Table 4. Calprotectin concentrations (CALP, µg/ml) according to mother's mass increase during pregnancy.

Mother's mass increase during pregnancy	<12 kg	12-18 kg	>18 kg	p/epsilon-squared
	Median (range)	Median (range)	Median (range)	
CALP in meconium	132.13 (7.48-733.43) n=20	157.90 (6.93-866.24) n=29	152.16 (11.74-884.11) n=23	0.450/ 0.02
CALP in newborn stool	95.86 (11.89-280.87) n=19	128.27 (35.76-627.35) n=28	151.50 (26.58-571.09) n=21	0.216/ 0.05
CALP in maternal stool	18.00 (17.60-24.31) n=3	46.36 (3.89-161.21) n=10	98.75 (74.79-211.77) n=7	0.031†/ 0.37

† <12 vs 12-18: p=0.877; <12 vs >18: p=0.047; 12-18 vs >18: p=0.145; †- test post-hoc; p - statistical significance, epsilon-squared – effect size

Supplemental Table 5. Zonulin concentrations (ZON, ng/ml) according to mother's BMI increase during pregnancy.

Mother's BMI change	>5.7	≤5.7	
	Median (range)	Median (range)	p/r
ZON in meconium	47.38 (1.36-309.20) n=24	63.53 (1.93-700.65) n=27	0.210/ -0.18
ZON in newborn stool	107.30 (50.97-505.51) n=28	133.51 (29.38-593.72) n=38	0.215/ -0.15
ZON in umbilical-cord serum	11.16 (5.82-47.92) n=38	11.14 (6.86-52.34) n=39	0.752/ -0.04
ZON in maternal serum	21.95 (9.28-57.54) n=37	21.37 (6.39-42.98) n=39	0.689/ 0.05
ZON in maternal stool	72.96 (42.52-225.74) n=15	114.56 (75.93-188.70) n=12	0.004/ -0.55

BMI – Body Mass Index, p - statistical significance, r-effect size,

Supplemental Table 6. Concentrations of calprotectin (CALP, μg / ml) according to mother's BMI increase during pregnancy.

Mother's BMI change	>5.7	≤5.7	
	Median (range)	Median (range)	p/r
CALP in meconium	170.52 (11.74-884.11) n=32	143.27 (6.93-737.25) n=41	0.523/0.07
CALP in newborn stool	153.35 (26.58-571.09) n=32	113.67 (23.27-627.35) n=37	0.030/0.26
CALP in maternal stool	98.75 (38.30-211.77) n=11	18.00 (3.89-121.13) n=9	0.003/0.66

BMI – Body Mass Index, p - statistical significance, r-effect size

Supplemental Table 7. Correlations between maternal BMI and zonulin concentrations (ZON).

BMI	Before pregnancy	Before birth	BMI change
ZON in meconium	R=0.14 n=51	R=0.02 n=51	R=-0.15 n=51
p	0.320	0.914	0.311
ZON in newborn stool	R=0.14 n=66	R=0.08 n=66	R=-0.10 n=66
p	0.279	0.532	0.442
ZON in maternal stool	R=0.03 n=28	R=-0.25 n=28	R=-0.63 n=27
p	0.892	0.197	0.0004
ZON in maternal serum	R=-0.21 n=76	R=-0.14 n=76	R=0.03 n=76
p	0.073	0.229	0.767
ZON in umbilical-cord serum	R=0.11 n=77	R=0.07 n=77	R=-0.06 n=77
p	0.354	0.532	0.584

BMI – Body Mass Index, p - statistical significance, R- Spearman correlation coefficient

Supplemental Table 8. Correlations between maternal BMI and calprotectin concentrations (CALP).

BMI	Before pregnancy	Before birth	BMI change
CALP in meconium	R=0.21 p=0.068 n=73	R=0.23 p=0.052 n=74	R=0.05 p=0.645 n=73
CALP in newborn stool	R=0.07 p=0.552 n=69	R=0.10 p=0.388 n=70	R=0.15 p=0.215 n=69
CALP in maternal stool	R=-0.07 n=20 p=0.779	R=0.13 n=20 p=0.589	R=0.59 n=20 p=0.006

BMI – Body Mass Index, R- Spearman correlation coefficient, p- statistical significance

Supplemental Table 9. Effect of birth weight on zonulin concentrations (ZON, ng/ml).

Birth weight	>15 percentile	≤15 percentile	
	Median (range)	Median (range)	p/r
ZON in umbilical-cord serum	11.12 (5.82-47.92) n=69	12.29 (7.48-52.34) n=10	0.621/ -0.06
ZON in meconium	58.63 (1.36-700.65) n=44	39.54 (8.14-148.24) n=8	0.630/ 0.07
ZON in newborn stool	121.16 (29.38-505.51) n=57	78.91 (55.03-593.72) n=11	0.150/ 0.17
ZON in maternal serum	20.98 (6.39-57.54) n=68	22.58 (11.09-42.98) n=10	0.525/ -0.07
ZON in maternal stool	80.97 (42.52-208.20) n=23	114.56 (56.81-225.74) n=6	0.247/ -0.21
Birth weight	≥85 percentile	≤15 percentile	
ZON in meconium	87.95 (7.40-122.11) n=6	28.91 (8.14-148.24) n=9	0.239/ -0.30
ZON in newborn stool	114.50 (88.24-505.51) n=7	78.91 (55.03-593.72) n=11	0.085/ -0.41
ZON in umbilical-cord serum	10.63 (5.82-16.82) n=11	12.70 (7.48-52.34) n=11	0.168/ 0.29
ZON in maternal serum	21.37 (14.49-39.28) n=11	23.76 (11.09-42.98) n=11	0.555/ 0.13

p - statistical significance, r-effect size

Supplemental Table 10. Effect of birth weight on calprotectin concentrations (CALP, µg/ml).

Birth weight	>15 percentile	≤15 percentile	
	Median (range)	Median (range)	p/r
CALP in meconium	154.76 (6.93-884.11) n=66	152.31 (11.43-733.43) n=10	0.695/-0.04
CALP in newborn stool	140.94 (11.89-627.35) n=62	108.52 (35.76-178.07) n=10	0.248/0.14
CALP in maternal stool	74.79 (3.89-211.77) n=17	54.42 (13.26-121.13) n=4	0.893/0.03
Birth weight	≥85 percentile	<15 percentile	
CALP in meconium	196.29 (11.74-884.11) n=10	161.35 (11.43-733.43) n=11	0.699/-0.08
CALP in newborn stool	172.04 (49.76-518.81) n=10	108.52 (35.76-178.07) n=10	0.162/-0.31
CALP in maternal stool	74.79 (58.49-101.83) n=3	54.42 (3.89-121.13) n=5	0.551/-0.21

p - statistical significance. r-effect size
